# Accurate Assessment of the Oxygen Reduction Electrocatalytic Activity of Mn/Polypyrrole Nanocomposites Based on Rotating Disk Electrode Measurements, Complemented with Multitechnique Structural Characterizations

**DOI:** 10.1155/2016/2030675

**Published:** 2016-11-30

**Authors:** Patrizia Bocchetta, Carolina Ramírez Sánchez, Antonietta Taurino, Benedetto Bozzini

**Affiliations:** ^1^Dipartimento di Ingegneria dell'Innovazione, Università del Salento, Via Monteroni, 73100 Lecce, Italy; ^2^Centro de Investigación, Innovación y Desarrollo de Materiales-CIDEMAT, Universidad de Antioquia UdeA, Calle 70 No. 52-21, Medellín, Colombia; ^3^Institute for Microelectronics and Microsystems, IMM-CNR, Via Monteroni, 73100 Lecce, Italy

## Abstract

This paper reports on the quantitative assessment of the oxygen reduction reaction (ORR) electrocatalytic activity of electrodeposited Mn/polypyrrole (PPy) nanocomposites for alkaline aqueous solutions, based on the Rotating Disk Electrode (RDE) method and accompanied by structural characterizations relevant to the establishment of structure-function relationships. The characterization of Mn/PPy films is addressed to the following: (i) morphology, as assessed by Field-Emission Scanning Electron Microscopy (FE-SEM) and Atomic Force Microscope (AFM); (ii) local electrical conductivity, as measured by Scanning Probe Microscopy (SPM); and (iii) molecular structure, accessed by Raman Spectroscopy; these data provide the background against which the electrocatalytic activity can be rationalised. For comparison, the properties of Mn/PPy are gauged against those of graphite, PPy, and polycrystalline-Pt (poly-Pt). Due to the literature lack of accepted protocols for precise catalytic activity measurement at poly-Pt electrode in alkaline solution using the RDE methodology, we have also worked on the obtainment of an intralaboratory benchmark by evidencing some of the time-consuming parameters which drastically affect the reliability and repeatability of the measurement.

## 1. Introduction

Notwithstanding the astonishing discrepancies among the ORR activities reported from different laboratories and typically attributed to experimental protocols, electrode conditioning, and current-potential curves corrections [1], the RDE technique—together with the RDDE one—seems to be the method most used by the electrochemists to characterize ORR electrocatalysts. The reasons are simplicity and speed of the measurements as well as commercial availability and low cost of all the required facilities. For this specific electroanalytic application, poly-Pt can be considered as a standard electrode that, notwithstanding the intrinsically poorly defined nature of a polycrystalline surface, exhibits a very robust and reproducible electrochemical response. Moreover, since standardized disks of poly-Pt are commercially available at convenient costs, their use as benchmark electrodes for the ORR electrocatalytic studies seems appropriate. At present, ORR at Pt RDE electrodes of different forms (wires, gauzes, foils, and disks of different areas) has been widely investigated in acid solution [1–3], due to their relevance in polymer electrolyte fuel cells [4]. On the contrary marginal attention has been paid to pure Pt in alkaline solutions (single crystal Pt [5, 6], poly-Pt particles [7], poly-Pt wire [8], poly-Pt thin film [9–11], and poly-Pt rod [6, 12–14]) despite the fact that ORR plays a key role in the chlor-alkali technology and in metal-air batteries [15, 16]. Due to the absence of an interlaboratory benchmark material in alkaline solution and the discrepancies in the few data available at the moment [6, 12–14], we have realized an intralaboratory poly-Pt benchmark against which the data gathered with novel ones can be safely gauged.

Limited interest for Pt as ORR electrocatalyst in alkaline solution is probably justified by high costs, less aggressive chemical conditions, and lower catalytic activity and efficiency due to HO_2_^−^ generation [17]. For these reasons, research in alkaline ORR has been centred on catalysts featuring nonnoble metals [18] such as palladium [19], ruthenium [20], iron [21, 22], nickel and cobalt mainly in the form of spinels [23–26], perovskites [27, 28], and manganese oxides [14, 29–36]. In particular, MnO_*x*_ are the most studied systems for applications in metal/air batteries [16, 37, 38] owing to their low cost, adequate electrochemical stability, and good catalytic activity towards the decomposition of HO_2_^−^ species produced by the ORR 2*e* ^−^ pathway in alkaline solution [30, 31]. One of the main limits of MnO_*x*_-based ORR electrocatalysts is their poor electrical conductivity: to overcome this problem commercial air cathodes have to be fabricated by combining oxides with conducting materials, mainly carbon nanoparticles [39]. Among other approaches favouring electronic contact to MnO_*x*_, the dispersion of metal oxide particles in a polypyrrole (PPy) matrix is also appealing for a series of physicochemical reasons, detailed in [40–43]. Among Mn/PPy fabrication routes, electrodeposition offers the possibility of forming directly Mn particles into the polymer layer [44, 45] or of entrapping metal complexes that yield metal particles during a subsequent reduction step [46]. The first studies that have demonstrated the possibility of increasing the mixed oxide conductivity by using PPy [40] also showed that the deposition of alternating layers of PPy and MnCu or NiCu oxide spinel particles protects the catalytic sites against dissolution during ORR operation. Recently, an alternative facile synthesis of composite metal oxide/PPy electrocatalysts has been proposed, based on one-pot electrodeposition onto conductive substrates [47–52].

In this paper we have focused on the quantitative and reproducible assessment of the ORR activity of electrodeposited Mn/PPy nanocomposites in aqueous alkaline ambient, by using poly-Pt electrodes as intralaboratory benchmark material. The electrocatalytic results are complemented by a morphological and structural characterization of our materials by FE-SEM, AFM, conductivity-probe SPM, and Raman Spectroscopy.

## 2. Materials and Methods

### 2.1. Materials and Electrodes

The Mn/PPy electrodeposition bath contains the following: 0.1 M pyrrole, 25 mM MnCl_2_, 0.1 M tetrabutyl-ammonium-perchlorate (TBAP), and 1% v/v H_2_O dissolved in acetonitrile. Acetonitrile, MnCl_2_·4H_2_O, pyrrole, and TBAP were supplied by Aldrich. Before each PPy electrodeposition experiment, the pyrrole monomer was distilled under rotary pump vacuum several times (typically three) until it becomes colourless. All the solutions were prepared with ultrapure water from a Milli-Q system (Millipore, Vimodrone (MI), Italy), exhibiting a resistivity of 18.2 M*Ω* cm. Polycrystalline-Pt (Poly-Pt) rod was mechanically polished with different grades of grit paper, down to 0.05 *μ*m diamond paste, and then accurately rinsed with double-distilled water. To guarantee an initial reproducible surface, the working electrode was held for 5 s at 1.40 V/RHE, a potential which a full coverage of platinum surface by O-containing species is attained to, followed by an electroreduction step to 1.13 V/RHE for 1 min [9]. The surface of the graphite (G) was treated according to the following procedure: (i) polishing to mirror finish; (ii) 10 min ultrasonication (US) in ultrapure H_2_O; (iii) repolishing to mirror finish; (iv) 10 min US in acetone US; and (v) 10 min US in ultrapure H_2_O. Electrochemical synthesis was performed using a three-electrode cell with a graphite disk (Φ = 1.35 mm), as working electrode (WE), a Pt wire as counter electrode (CE), and an aqueous silver/silver chloride (Ag/AgCl/3 M: 0.209 V/NHE) as reference electrode (RE). All potentials are reported versus the reversible hydrogen electrode (RHE) scale.

### 2.2. Electrochemical Measurements and Methods

The electrochemical measurements were carried out at room temperature using a ParStat 2273 potentiostat. The Mn/PPy catalysts were synthesized in deaerated electrodeposition baths. Electrodeposition was carried out with the pulsed potential procedure described in previous works [47, 48, 50, 51] optimized in view of growing a composite consisting of two constituents that are formed anodically (PPy and Mn(III, IV) compounds) and cathodically (Mn(0) and/or precipitated Mn(II) containing species). The initial step (0.971 V/RHE) does not lead to faradaic reactions, but it is required to relax the compositional double layer. After this relaxation step, a layer of PPy is electrodeposited during the first anodic pulse (2.17 V). During the subsequent cathodic pulse (0.829 V), reduced Mn(0) or precipitated Mn(II) species can be incorporated into PPy. In the final anodic step of each cycle (2.17 V), another layer of—possibly Mn-doped—PPy is deposited. After electrochemical synthesis, the films were rinsed with pure acetone and dried at room temperature.

### 2.3. Morphological Characterization

The morphology of Mn/PPy catalyst electrodeposited onto graphite discs was investigated by Field-Emission Scanning Electron Microscopy (FE-SEM) using an NVISION 40 Zeiss, equipped with a high resolution Gemini Field-Emission Gun and with an Oxford INCA 350 Xact Energy Dispersive X-Ray (EDX) Spectrometer. Details about the morphological features at the nanoscale and quantitative analysis of roughness and surface area were obtained with a Nanoscope III Multimode Atomic Force Microscope (AFM) (Bruker) in air using the ScanAsyst mode and the ScanAsyst probe (Bruker). On each sample 5 different square areas of sizes 1, 5, and 20 *μ*m were scanned at rates of 0.7 and 1.5 Hz. The electrical conductivity of the sample was mapped by the PeakForce TUNA Module (Bruker) using a Pt/Ir probe (PF-TUNA 0.4 N/m, Bruker). AFM image analysis was carried out with the Nanoscope Analysis 1.5 program (Bruker).

### 2.4. Raman Spectroscopy

Raman spectra were recorded using a LabRam microprobe confocal system. A 10x long-working distance objective was used and the excitation line at 632.8 nm was provided by a 12 mW He-Ne laser. The slit and pinhole were set at 200 and 400 *μ*m, respectively, corresponding to a scattering volume of ~3 pL; Raman spectra were acquired with a 600 grid/mm spectrometer. The recorded Raman intensities are directly proportional to the discharge current of the CCD detector.

### 2.5. ORR Electrocatalytic Measurements

Oxygen reduction reaction (ORR) linear sweep voltammetries (LSV) on Mn-PPy/C electrodes were recorded to evaluate the catalytic activity in an O_2_-saturated (SIAD 6.0) 0.1 M KOH electrolyte at quasi-steady-state (5 mV s^−1^) at different Rotating Disk Electrode (RDE) rotation speeds. Graphite disks with Mn/PPy electrodeposit were mounted in a Rotating Disk Electrode System (ParStat Model 2273, Photo Analytical S.R.L., Settala (MI), Italy). The same electrochemical experiments were duplicated with solutions that had been deoxygenated by N_2_ saturation. The current densities are referred to the electrode geometric area (1.326 cm^2^). O_2_ was bubbled for 20 min into the solution before the measurements and an O_2_ blanket was kept above the electrolyte during the measurement. In order to minimize disturbing effects of bubbles during the experiments, the electrolyte saturation has been performed at open circuit for 20 min and the pipette allowing gas into the solution was raised during the RDE measurement leaving a gas blanket over the solution. Special care was taken to avoid contingent ambient air suction into the analysis compartment caused by the electrode rotation. The ORR Levich slopes were estimated from current-voltage curves which the N_2_-background had been subtracted from.

## 3. Results and Discussion

### 3.1. The RDE Measurement

#### 3.1.1. Theoretical Aspects

**Figure 1:**
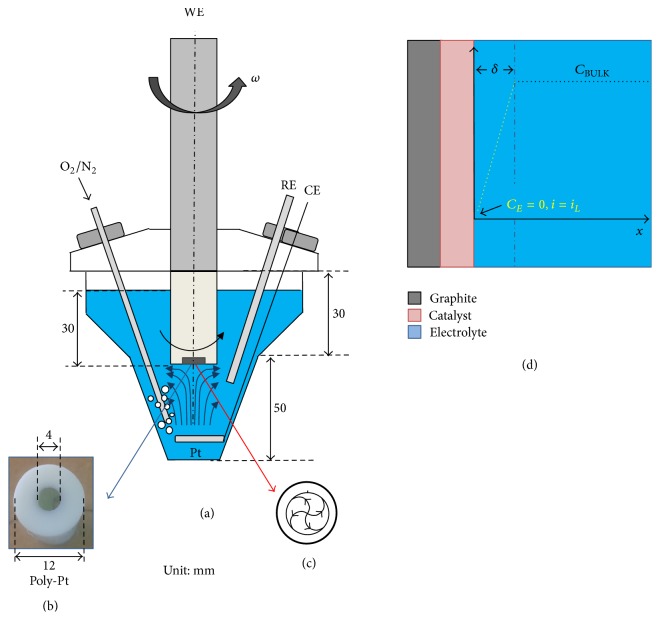
(a) Scheme of the electrochemical cell used for the ORR RDE experiment. (b) Macrograph of the poly-Pt electrode used for benchmark tests. (c) Representative flow lines on the electrode surface under rotation. WE, RE, and CE indicate working, reference, and counter electrodes, respectively. (d) Sketch of the catalyst-electrolyte interface depicting the linear O_2_ diffusion model that is incorporated in the K–L equation. The dotted line represents the O_2_ concentration distribution both in bulk electrolyte (black) and in the boundary layer (yellow). *δ* is the boundary layer thickness; *C*_BULK_ and *C*_*E*_ are the O_2_ concentrations in the bulk and at the solution | catalyst surface, respectively; *i*_*L*_ is the O_2_ diffusion-limiting current.

As it is well known from the literature [53], RDE is an electrochemical technique based on controlled electrolyte convection, leading to a well characterized fluid velocity distribution on the working electrode, provided that appropriate boundary conditions are implemented in cell and electrode mounting as well as in the real shaft rotation process. In the RDE method a discoidal electrode (e.g., glassy carbon or platinum), embedded in a rod of an insulating material (e.g., glass or Teflon), connected to a motor, rotates at a certain frequency *f* = *ω*/2*π*, where *ω* is the angular velocity (Figures 1(a) and 1(b)). The RDE technique operates on the principle of controllable stationary limiting currents, where the diffusion layer at the electrode maintains its thickness constant over time. To this end, the effective electrode surface and the scan rate are kept to values in correspondence of which double-layer charging produces a vanishing current. Under these hypotheses, the mass transfer process can be described with simple equations that allow correlating the experimental parameters to the mass transfer of reactants to the electrode surface. When an electrode is rotated, mass transfer of reactants and products is governed by convective-diffusion mechanisms. At the RDE the hydrodynamic flow pattern results from centrifugal forces that move the liquid horizontally out and away from the centre of the disk (Figure 1(c)) while fresh solution continually replaces it by a flow normal to the electrode surface (Figure 1(a)). Of course, for these condition to hold true, a set of conditions have to be met, the details of which are provided, for example, in [53].

The convective-diffusion equations imply the existence of a diffusion layer of quiescent electrolyte on the electrode surface, within which mass transfer takes place only by diffusion. Outside this layer of thickness *δ*, the convection maintains the concentrations of all species uniform and equal to the bulk values (Figure 1(d)). According to this model, by introducing the adjustable parameter *δ*, the convection problem is formally treated as a diffusional one [54]. O_2_ diffusion to the catalyst surface may thus be modelled as illustrated in Figure 1(d): O_2_ diffuses through the boundary layer (*δ*) in the electrolyte solution yielding a controlled O_2_ flux/limiting current. In the relevant geometry the steady-state convective-diffusion equation with mass-transport controlled source term can be solved analytically, yielding the Koutecky–Levich (K–L) equations [54]:

(1) 1j=1jL+1jK=1B·ϖ+1jK,

(2) B=0.62·n·F·Co·Do2/3·v−1/6,

where *j*_*K*_ is the kinetic current density; *j*_*L*_ the diffusion–limiting current density; *j* the measured current density; *B* the reciprocal of the slope; *ϖ* the angular velocity of the disk; *F* the Faraday constant; *C*_0_ the saturation concentration of O_2_ in 0.1 M KOH at room temperature (1.2 10^−6^ mol cm^−3^); *D*_0_ the diffusion coefficient of oxygen in water (1.73 10^−5^ cm^2^ s^−1^); *v* the kinematic viscosity of the solution at room temperature (0.01 cm^2^ s^−1^) [55]; and *n* the electron transfer number.

#### 3.1.2. Methodological Aspects

**Figure 2:**
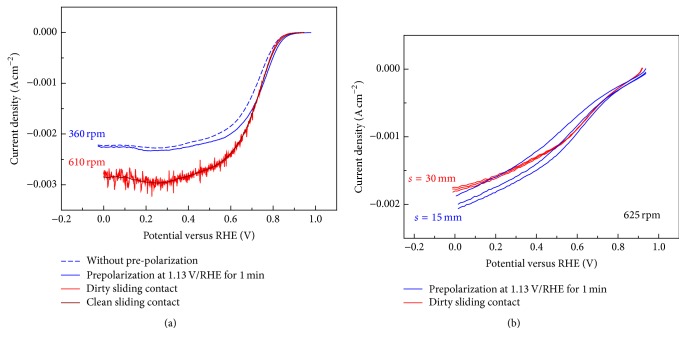
RDE voltammograms (scan rate: 5 mV s^−1^) in O_2_-saturated 0.1 M KOH evidencing the effect of the following: poly-Pt electrode before polarization (blue curves) and quality of the RDE sliding electric contacts (red curves) (a) and *s* parameter of the cell design for Mn-PPy240/G (b). The N_2_ background has not been subtracted.

The literature survey on ORR at poly-Pt electrode in alkaline solution using the RDE methodology is limited and not consistent [6, 12–14]; therefore a definite comparison between the new developed Mn-PPy material and the state-of-the-art electrocatalysts is not allowed. As a consequence, we have worked to obtain an intralaboratory benchmark (poly-Pt rod) not aiming at proposing a standard RDE curve usable as reference for general activity of poly-Pt (to do that a dedicated work of analysis on the different parameters affecting the ORR activity is necessary, together with interlaboratory testing; this kind of work is actually a challenge especially in alkaline solution) but only at acquiring an internal reference on the basis of which our novel data can be safely evaluated. In Section 3.1.2's (1) and (2) the effects of (i) the distance (*s*) between the catalytic surface and the electrolyte-free surface and (ii) the initial features of the catalyst surface on the reliability and reproducibility of the ORR measurements at RDE are discussed. Other experimental issues, such as glassware electrolyte impurity levels and uncompensated electrolyte resistance, slightly affect the results with errors less than the 2%, while dirty sliding electric contact of the rotator is responsible for a strong increase in the signal-to-noise ratio of the LSV curve (Figure 2(a), red curves).

(1*) Electrochemical Cell Design*. The electrochemical cell design significantly affects the hydrodynamic conditions of the electrolyte that must satisfy the above mentioned conditions (Section 3.1.1) to allow quantitative analysis according to the Levich equation. The cell and experimental conditions must be therefore designed so that the solution flow within the cell becomes laminar. In this work, we report a detailed cell design (see the scheme reported in Figure 1(a)) associated with the RDE voltammetric curves of ORR at poly-Pt and Mn/PPy catalysts in 0.1 M KOH. In our experiments, we have evidenced that the determination of the distance (*s*) between the catalytic surface and the electrolyte-free surface (i.e., the electrolyte volume) radically improves the quality of RDE voltammetries due to achieved laminar flow at the reacting electrode surface. The optimal *s* value was determined by experimental calibration and found to be 30 mm, corresponding to a volume of the electrolyte of 80 mL (Figure 2(a)). In Figure 2(b) it can be observed that the effect of *s* on the LSV curve is to modify (i) the Butler-Volmer behaviour under mixed kinetic control in the intermediate potential region and (ii) the curve repeatability. In Table 1 the scatter of current density values (standard deviation) obtained from repeated measurements at the same rotation rates is shown. Low *s* values find out a low reproducibility because, due to the turbulence effects, the diffusion limited conditions are neither constant nor controllable during the potential scan. As a consequence, the limiting current determination is not possible without making fundamental errors. 

(2*) Initial Electrode Conditions*. It is well known that the state and coverage of oxygen-containing species on platinum are a crucial factor in determining the electrocatalytic activity of platinum surfaces towards O_2_ reduction [9]. For this reason, the reproducibility of the initial surface of poly-Pt was assured by holding the electrode for 5 s at 1.40 V/RHE, a potential which a full coverage of platinum surface by O-containing species is attained at, followed by an electroreduction step at 1.13 V/RHE for 1 min [9]. The effect of this pretreatment reported in Figure 2(a) (blue curves) is to shift the onset and half-wave potential to more positive values. At low overpotentials the surface of untreated Pt is covered of oxygen intermediates strongly bonded with Pt that affect the activation energy of the ORR process, hindering the proton and electron transfer, while at high overpotentials their influence becomes negligible because the stability of the oxygen bond decreases and the ORR may proceed (the two curves overlap).

The surface initial conditions of Mn/PPy electrocatalyst are strictly related to the morphochemical goodness of the electrodeposit; this aspect is thoroughly discussed in Section 3.3 where the morphological and molecular structure of Mn/PPy materials has been characterized by FE-SEM, AFM, conductivity-probe SPM, and Raman Spectroscopy in view of ORR performance evaluation.

### 3.2. The RDE Experiment at Poly-Pt Electrode in 0.1 M KOH

**Figure 3:**
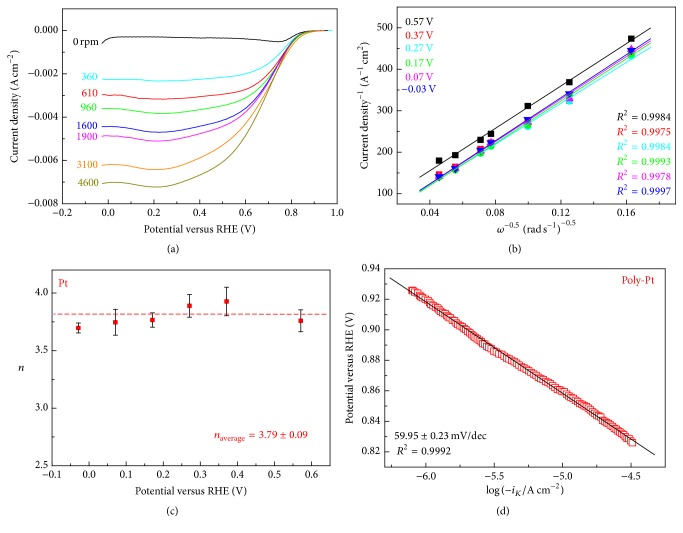
(a) RDE voltammograms (scan rate: 5 mV s^−1^) of poly-Pt electrode in O_2_-saturated 0.1 M KOH at different rotation speeds. The N_2_ background has been subtracted. (b) Koutecky–Levich plots derived from the curves of Panel (a) at a series of representative potentials. Symbols are experimental data; lines are the linear regressions. (c) Dependence of the electron transfer number *n* on the electrode potential. (d) Tafel plot obtained from RDE voltammograms at 1600 rpm.

Poly-Pt rod has been used as intralaboratory benchmark material in alkaline solution for the study of ORR at Mn-PPy/G catalyst. The electrode (Figure 1(b)) has been pretreated using the protocol described in Section 3.1.2.(2). In Figure 3(a) we report ORR quasi-steady-state voltammograms of poly-Pt rod at a scan rate of 5 mV s^−1^ in O_2_-saturated 0.1 KOH aqueous solutions at different RDE rotation speeds. Stable and reproducible voltammograms were obtained following the experimental details discussed in Section 3.1. At low overpotentials (0–0.2 V/RHE) the curves are independent of the rotation speed, *ω*, being the electrode kinetics governed by the charge transfer. At higher overpotentials instead, the increase of current density with potential becomes slower, as expected for an electrochemical reaction under mixed control, until the mass-transport kinetic contributions become significant and determine the appearance of a current plateau, which thus strongly depends on *ω*. Sometimes a slight hump at 0.20 V is observed and presumably attributed to H-adatom adsorption on platinum [13]. In agreement with the Levich equation (see (2)) the height of this plateau increases linearly with the square root of *ω* as shown in Figure 3(b). The slopes of the linear fit lines to the experimental Levich plots were used to estimate *n* according to (1) in a range of electrode potentials that are representative of practical electrocatalytic operation and reported in Figure 3(c). *n* values, averaged in the potential range −0.6 ÷ −0.1, were found to be 3.79 ± 0.09, suggesting the prevalence of ORR four-electron mechanism typical of platinum.

The charge-transfer kinetic is shown in the Tafel plots of Figure 3(d), where ORR kinetic current *i*_*k*_ is estimated according to 1/*i* = 1/*i*_*L*_ + 1/*i*_*k*_ (*i*_*L*_: limiting current density). The Tafel slope is approximately 60 mV dec^−1^ in agreement with literature for Pt under alkaline conditions (0.1–6.0 M KOH) in the low overpotential region [10–13, 56]. This value, close to −2.303*RT*/*αF* ≈ 60 mV/dec (where *R* is the molar gas constant, *T* the absolute temperature, *α* the transfer coefficient, and *F* the Faraday constant) at room temperature [57, 58], indicates that the transfer of the first electron catalysed by Pt is probably the rate-determining step.

### 3.3. Characterization of Mn-PPy/G ORR Catalyst

#### 3.3.1. Morphological and Structural Characterization

**Figure 4:**
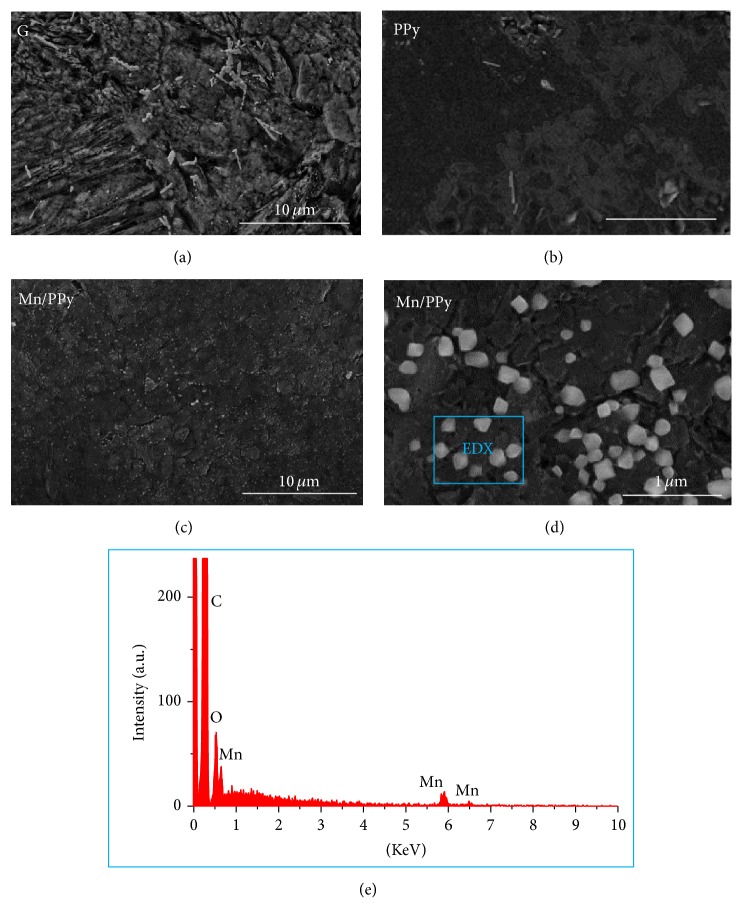
FE-SEM micrographs of (a) graphite, (b) PPy (PPy60/G), and (c, d) Mn/PPy electrodeposited on graphite (Mn-PPy240/G). (e) A typical EDX spectrum recorded in a representative area.

The morphological, compositional, and chemical-state distribution of the as-deposited Mn/PPy material, using the same electrodeposition procedure employed for this investigation, has been studied in detail by soft X-ray fluorescence (XRF) mapping and microabsorption spectroscopy (micro-XAS) in a previous paper of ours [51]. This analysis disclosed a rather homogeneous Mn spatial distribution that is consistent in a rather wide range of current densities, while micro-XAS has revealed that the potentiostatic pulsed codeposition process—alternating anodic and cathodic polarization intervals—leads to a PPy deposit containing manganese (II, III, IV) oxyhydroxides and traces of Mn(0). A granular morphology was found in all the analyzed regions, with colocation of Mn, N, and O, denoting the fact that MnO_*x*_ and polypyrrole act as nucleation centres for material electrodeposited in both the anodic and cathodic pulses. This is in agreement with the classical nucleation and growth mechanism for electrodeposition by pulse-plating [59]. In this paper, we have assessed the morphological structure of the G substrate, of a pure PPy deposit after 60 electrodeposition cycles (PPy60/G), Mn/PPy after 60 cycles (Mn-PPy60/G), and Mn/PPy after 240 cycles (Mn-PPy240/G).

(1*) FE-SEM and EDX Analyses*. FE-SEM analysis has been used to assess the distribution of the deposit over the whole electrode area.  Figure 4 reports the in-plane morphology at low magnification of the G, PPy/G, and Mn-PPy240/G samples. The G substrate, treated as specified in Section 2.1, shows a highly structured morphology with a high surface area (Figure 4(a)); the electropolymerization of PPy leads to a film covering uniformly the G electrode (Figure 4(b)) while a uniform distribution of Mn-containing nanoparticles is disclosed after electrodeposition of Mn/PPy (Figures 4(c) and 4(d)). The presence of Mn has been positively assessed by EDX analysis, a typical spectrum is shown in Figure 4(e).

(2*) AFM and PF-TUNA Analysis*. AFM analysis has been used to obtain quantitative morphological information on the surface: average grains size (*R*_*Q*_), average roughness (*R*_*A*_), and the real surface area (data summarized in Table 1). PF-TUNA analysis has been also performed to map the electrical conductivity.

**Figure 5:**
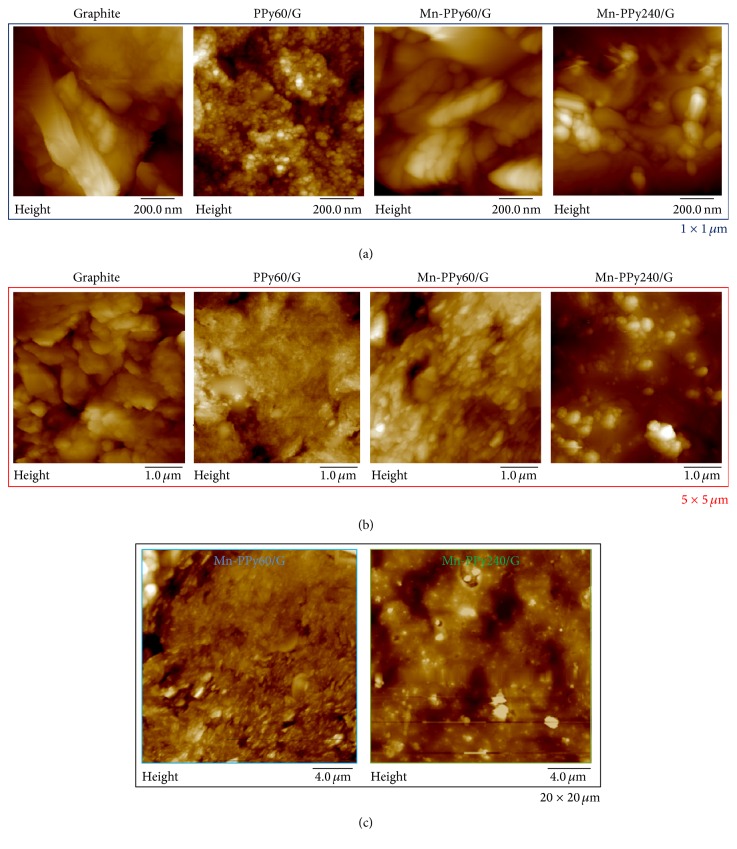
AFM height images of the graphite (G) substrate as well as of the following: PPy60/G, Mn-PPy60/G, and Mn-PPy240/G electrodeposits.

Figure 5 reports AFM height images of G substrate and of the following electrodeposits: PPy60/G, Mn-PPy60/G, and Mn-PPy240/G. The AFM images of graphite substrate confirm the presence of a highly structured morphology and provide quantitative information on the roughness and surface area (Table 2). The electrodeposition of PPy for 60 cycles to cover the graphite surface with a closely packed population of globules having a mean diameter of 21 ± 5 nm: a concomitant reduction of the surface area by ca. 25% is recorded after the electropolymerization process (Table 2). The PPy globular structure shown in Figure 5(a) is typical for PPy thicknesses below 1 *μ*m [60], such as in our sample, the mean thickness of which (*δ*) has been estimated to be 0.9 *μ*m (in good agreement with literature [61]) from the electrical charge (*Q*) associated with pyrrole oxidation by application of Faraday's law and assuming 100% current efficiency for polypyrrole formation according to *δ* = *QM*/*PAzF*, where *M* is the molar mass of the repetitive unit (67.09 g mol^−1^), *F* the Faraday constant (96,500 C mol^−1^), *Q* the electrical charge, *A* the working electrode area (1.326 cm^2^), *P* the density of the polymer (1.5 g cm^−3^), and *z* the electron loss (2.25) [62, 63]. By adding Mn^2+^ ions to the pyrrole-containing electrolyte and using the same number of electrodeposition cycles (60), the electrodeposition process is no longer able to produce a polymer film covering homogeneously the whole electrode surface. The AFM height images of the Mn-PPy60/G show that the surface is irregular with some uncovered areas and a roughness value that is intermediate between those of G and PPy60/G (Table 2). Of course, the morphology and metal distribution of Mn/PPy composites are affected by a wide range of parameters in a complex way, such as the electronic conductivity of PPy, doping degree, polymer porosity, metal ion transport within the PPy network structure, and chemical interactions between metal ions and their complexes with the polymer. This is chiefly instanced by the spread in *R*_*Q*_ values shown in Table 2, since this parameter is the most sensitive to the presence of peaks and valleys among the selected morphology indicators [64]. The morphology of electrodeposited Mn/PPy films improves by increasing the number of cycles to 240: in fact sample Mn-Py240/G is notably more compact and uniform coherently with SEM analysis (Figures 4(c) and 4(d)). In the AFM height images of Figure 4(c) the comparison between the samples Mn-PPy60/G and Mn-PPy240/G can be better appreciated at low magnification (20 × 20 *μ*m^2^). The thicker sample shows the presence of particles with diameters of 120 ± 81 nm, uniformly distributed over the whole surface. It is worth noting that the surface area of Mn-PPy240/G and that of the film-covered areas of Mn-PPy60/G are similar. The quality of the Mn-PPy240/G deposit is coherent with the superior ORR performance, discussed in Section 3.4.

**Figure 6:**
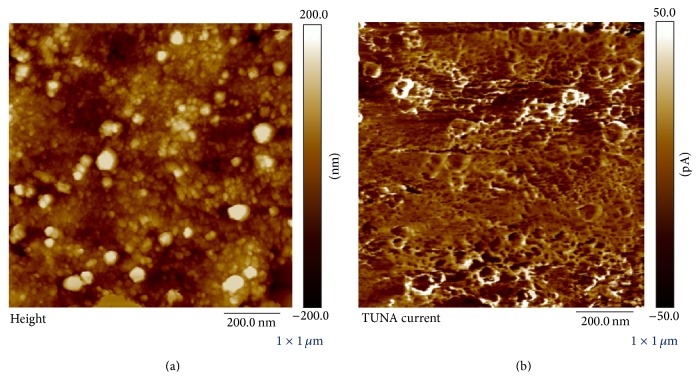
Simultaneous mapping of (a) topography and (b) current of Mn-PPy240/G electrodeposit obtained with PeakForce TUNA.

**Figure 7:**
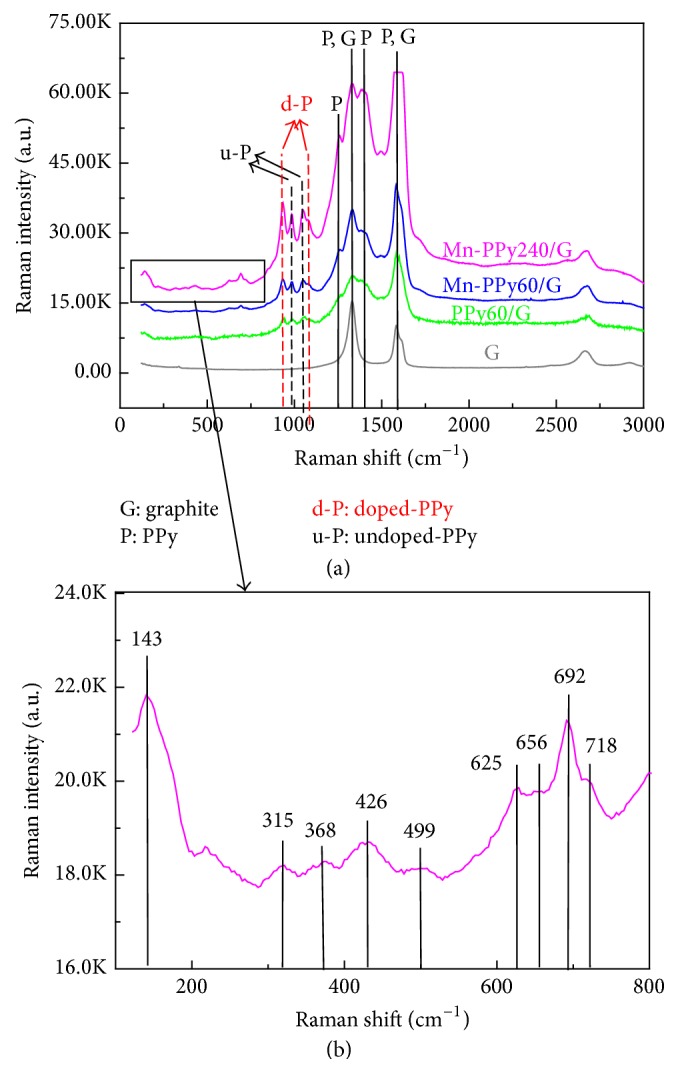
(a) Micro-Raman spectra of the graphite support (G) and of PPy60/G, Mn-PPy60/G, and Mn-PPy240/G electrodeposited catalysts. (b) Magnification in the range 100–800 cm^−1^ to highlight the MnO_*x*_ features.

Figure 6 shows typical topography (a) and current (b) images obtained simultaneously from the surface of the Mn-PPy240/G film. The current image clearly shows that the nanocomposite exhibits heterogeneous surface conductivity. By comparing Figures 6(a) and 6(b) one can straightforwardly conclude that the top regions of the particles are not conductive while current preferentially flows at the border of the particles. This finding suggests that the grain can be composed of poorly conductive manganese oxides, with PPy in the reduced state, coherently with XRF mapping and micro-XAS analysis [51] as well as Raman Spectroscopy (see next section). Moreover, our results are in keeping with a previous study, showing that the top of PPy globules is preferentially reduced during electrodeposition [60].

(3*) Raman Spectroscopy*. Micro-Raman Spectroscopy has been employed to characterize the PPy60/G, Mn-PPy60/G, and Mn-PPy240/G samples. In Figure 7(a) the Raman spectra of all PPy-containing samples display the typical prominent PPy bands [48]. Two of the three characteristic bands associated with the reduced/undoped (994, 1052 cm^−1^) and to the oxidized/doped form (938, 1090 cm^−1^) of PPy can also be appreciated. The other bands at 1564 cm^−1^ (reduced) and 1612 cm^−1^ (oxidized) are overwhelmed by the graphite signal. As is well documented in literature, the oxidation/reduction processes of PPy involve the fully reduced state (PP^0^), the oxidized state bipolaron (PP^2+^) and the intermediate oxidized state polaron (PPy^+•^) according to PPy^0^↔PPy^+•^↔PPy^2+^. The bands located at about 994 and 938 cm^−1^ belong to ring deformations associated with bipolaron (dication) and polaron (radical cation), respectively [65, 66], while the two peaks revealed at about 1090 and 1052 cm^−1^ can be attributed to the N–H in-plane deformation of oxidized and reduced polypyrrole state [67]. In the composite Mn/PPy samples some peaks appear in the range 100–800 cm^−1^ and become more intense in the thicker sample, where the Raman signal increases presumably due to the SERS effect discussed in [49]. According to the literature, the bands in the range 200–500 cm^−1^(see also Figure 7(b)) can be attributed to the Mn–O–Mn bending mode in the MnO_2_ lattice and those in the range 500–700 cm^−1^ to the Mn–O stretching of octahedral MnO_6_ groups [68–71]. The main band at 692 cm^−1^ is typically attributed to Mn_2_O_3_ [71], while the 625 cm^−1^ feature is reported as the main band of *β*-MnO_2_ [71] and the one at 143 cm^−1^ is reported to be the B1g mode of rutile-type *β*-MnO_2_ [71]. The concomitant presence of the four features at 315, 368, 499, and 656 cm^−1^ is usually ascribed to Mn_3_O_4_ [71, 72], but some of these bands can also appear in MnO_2_ [71, 73], Mn_2_O_3_ [72], or MnO [72, 74]. Even though the positions of Raman bands alone are not enough to support a conclusive speciation of Mn oxides not only owing to the intrinsic complexity of these vibrational spectra [68] but also owing to inconsistent literature in the field [75, 76], our Raman results, in combination with previous micro-XAS data [51], definitively prove that the electrodeposition process yields a mixture of Mn(II, III, IV) oxides.

### 3.4. The RDE Experiment at G, G/PPy, and Mn-PPy/G Electrodes in 0.1 M KOH

**Figure 8:**
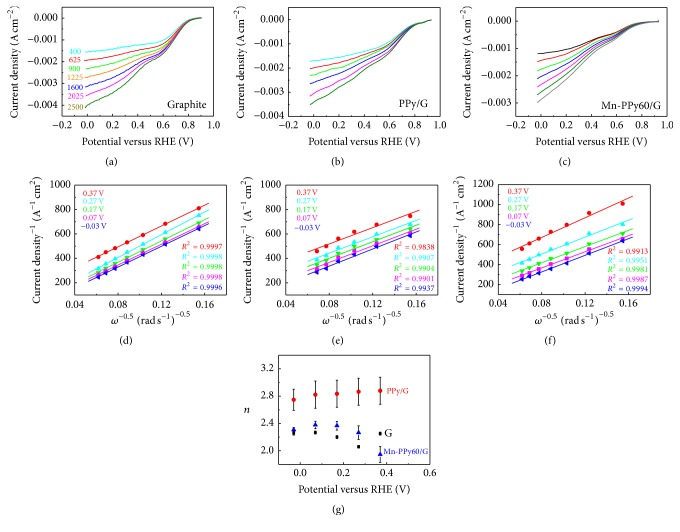
RDE voltammograms (scan rate: 5 mV s^−1^) in O_2_-saturated 0.1 M KOH at different rotation speeds of (a) G, (b) PPy/G, and (c) Mn-PPy60/G electrodeposits. The N_2_ background has been subtracted. (d–f) Koutecky–Levich plots derived from the curves of Panels (a, b, c) at a series of representative potentials. Symbols are experimental data; lines are the linear regressions. (g) Dependence of the electron transfer number *n* on the electrode potential.

**Figure 9:**
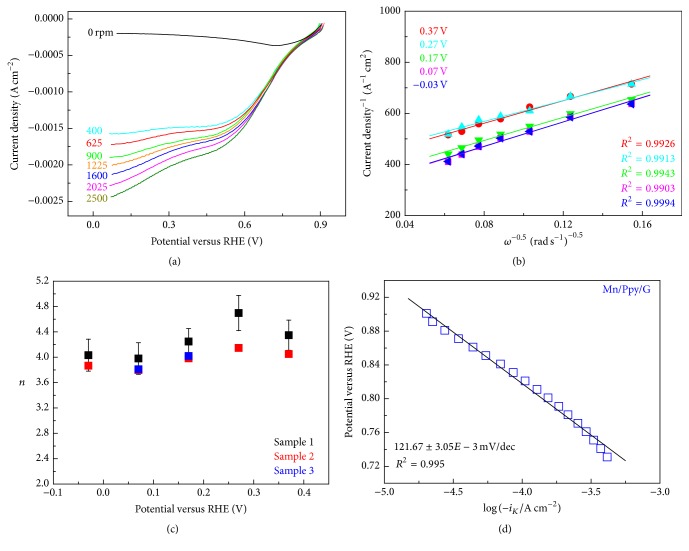
(a) RDE voltammograms (scan rate: 5 mV s^−1^) of Mn-PPy240/G catalyst in O_2_-saturated 0.1 M KOH at different rotation speeds. The N_2_ background has been subtracted. (b) Koutecky–Levich plots derived from the curves of Panel (a) at a series of representative potentials. Symbols are experimental data; lines are the linear regressions. (c) Dependence of the electron transfer number *n* on the electrode potential for three replicated samples. (d) Tafel plot obtained from RDE voltammograms at 1600 rpm.

The ORR electrocatalytic activity of the G substrate and that of electrodeposited PPy/G and Mn-PPy/G films have been evaluated through RDE measurements at different rotation speeds in 0.1 M KOH solution (Figures 8 and 9). The RDE steady-state voltammograms shown in Figures 8(a)–8(c) and 9(a) have been replicated three times at every rotation speed to check the electrode stability. In order to evaluate the catalytic efficiency comprehensively, electrocatalytic figures of merit have been estimated from RDE polarization curves obtained at 1600 rpm. The G substrate shows the two typical ORR waves reported for graphite in alkaline conditions [77]. In particular, the RDE polarization curve reported in [78] for bare graphite in an O_2_-saturated 0.1 M KOH solution at 1600 rpm fits very well with our curve reported in Figure 8(a). The Koutecky–Levich plots (Figure 8(d)) at different electrode potentials display good linearity and the slopes are constant over the potential range from 0 to 0.9 V/RHE, providing an electron transfer number around 2.3, independent of potential. This finding suggests that ORR on graphite in alkaline solution proceeds principally by the two-electron transfer to give peroxide ions and that no further reduction of HO_2_^−^ occurs, as generally accepted in literature [77, 79]. The current density in the LSV curves shown in Figure 8(a) has been calculated considering the geometric area of the graphite electrode; we have not considered the effective surface area estimated by AFM (Table 2) because for ORR electrocatalysts the impact of pore diameter can be more important than that of surface area [80] since micropores can be inactive [81].

The RDE curves recorded with PPy60/G and Mn-PPy60/G electrodes show a double wave structure that is less pronounced than the one observed with the bare graphite electrode (Figures 8(b), 8(c), and 9(a)). With the increasing of the rotation speed, the diffusion limited current plateau becomes inclined and this slope increases. This behaviour is quite common in the literature for carbon-based catalysts (pyrolyzed Mn, Co, Fe/PPy [47], Ag–MnO_2_ [82], pyrolyzed Fe(III)-tetramethoxyphenyl porphyrin [21], pyrolyzed cobalt [83] and iron phthalocyanine [84], and N-doped carbon nanotubes [85]) and has been attributed to the a heterogeneous distribution of the active sites on the surface catalyst [86, 87]. The ORR performance of our PPy60/G film is superior to that of PPy prepared by a polymerization process via chemical oxidation with FeCl_3_ [88]: both the onset (*E*_onset_) and half-wave (*E*_1/2_) potentials shift to more positive values, from 0.72 to 0.85 V/RHE and from 0.34 to 0.69 V/RHE, respectively. Moreover, the current density at 0.5 V/RHE increases from 1.8 to 2.2 mA cm^−2^. The K–L results (*n* = 2.74, Table 2) suggest that the O_2_ reduction at PPy60/G follows a combination of 2*e* ^−^ (reduction of O_2_ to HO_2_^−^) and 4*e* ^−^ (O_2_ is directly reduced to OH^−^) reactions. Coherently with the rather heterogeneous morphology observed by AFM (Section 3.3.1's (2)), the ORR performance of the Mn-PPy60/G catalyst is not significantly improved with respect to that of the Mn-free PPy60/G sample and the number of exchanged electron returns to a value of 2.25, similar to that of graphite, while the current density decreases notably (Table 2). The RDE analyses conducted on the Mn-PPy240/G sample, exhibiting the best morphology, are reported in Figure 9. The Koutecky–Levich plots and the correspondent *n* values at selected potentials (Figures 9(b) and 9(c)) indicate that 4*e* ^−^ oxygen reduction process is favoured similarly to poly-Pt electrode in the same ambient. In addition, *E*_onset_ and *E*_1/2_ potentials increase with respect to all the other samples reaching values very similar to poly-Pt (Table 3) and carbon/PPy materials [89]. On the contrary, the current density of the Mn-PPy240/G sample in the whole potential range analyzed is lower with respect to the benchmark poly-Pt. The expected lower catalytic activity of Mn/PPy with respect to poly-Pt is also evidenced in the Tafel plots of Figures 3(d) (poly-Pt) and 8(d) (Mn-PPy240/G). The Tafel slope for the Mn-PPy240/G is ca. 122 mV dec^−1^, notably higher than that found for ploy-Pt: ca. 50 mV dec^−1^, and suggests an electron transfer mechanism with adsorption molecular oxygen as the rate-determining step [47]. These values are in agreement with the literature, reporting values close to 120 mV dec^−1^ for Mn oxides (114 mV dec^−1^ for *γ*-MnO_2_/C [90], 120 mV dec^−1^ for high Mn_*x*_O_*y*_ load/C [91]).

## 4. Conclusions

Poly-Pt has been characterized as intralaboratory benchmark material for the assessment of the ORR electrocatalytic activity of Mn/polypyrrole nanocomposites based on RDE measurements. Accurate electrocatalytic data of Mn/PPy film—complemented with structural and morphological characterizations (FE-SEM, AFM, and Raman)—have been used to disentangle intrinsic electroactivity factors from surface coverage effects and have allowed optimizing the electrodeposition process parameters in view of global electrocatalytic activity. In particular, Mn/PPy grown with 240 electrodeposition cycles leads to the formation of continuous film catalytic particles ca. 120 nm in diameter. AFM PF-TUNA current imaging shows that the top region of these particles—probably consisting of MnO_*x*_ and reduced PPy—are poorly conductive and that the current preferentially flows at their border, where—owing to the prevailing current density distribution—polypyrrole is mainly in the oxidized state. These results are coherent with previous XRF mapping and micro-XAS analysis and with Raman Spectroscopy results reported in this paper that confirm the presence of the characteristic bands associated with the reduced/undoped and with the oxidized/doped form of PPy and of Mn(II, III, IV)O_*x*_. High-quality Mn/PPy electrodeposits exhibit a superior electrocatalytic activity due to higher four-electron-transfer selectivity for the ORR in alkaline solution, with respect to materials of the same chemistry grown suboptimal electrodeposition parameters. Moreover, optimized Mn/PPy exhibits electrocatalytic figures of merit such as *E*_onset_ and *E*_1/2_ potentials that are similar to those of poly-Pt, even though at lower nominal current densities.

## Figures and Tables

**Table 1:** Repeatability of RDE voltammograms (scan rate: 5 mV s^−1^) at Mn/PPy catalyst in O_2_-saturated 0.1 M KOH evaluated at 625 rpm and two *s* values.

Applied potential, V/RHE	Current density, mA cm^−2^			
	*s* = 15 mm		*s* = 30 mm	
	Average value	Standard deviation	Average value	Standard deviation
0.8	0.29627	0.032422	0.2841	0.001082
0.6	0,8248	0.125753	0.8803	0.005356
0.4	1.3697	0.142935	1.3090	0.016000
0.2	1.7189	0.114434	1.5957	0.019553

**Table 2:** AFM surface morphology indicators of graphite, PPy, and Mn/PPy samples.

Sample	Ideally flat	Graphite (G)	PPy on graphite (PPy/G)	Mn/PPy on graphite (Mn-PPy60/G)	Mn/PPy on graphite (Mn-PPy240/G)
Particle size/nm	/	/	21.11 ± 5.17	215.96 ± 125.27	120.05 ± 81
*R*_*A*_^*∗*^/nm	0	60.6 ± 5.94	25.3 ± 0.14	100.13 ± 64	76.03 ± 61.26
*R*_*Q*_^*∗∗*^/nm	0	80.7 ± 15.13	35.3 ± 1.56	133.6 ± 82.27	98.50 ± 78.95
Surface area/*μ*m^2^	100	148.6 ± 11.4	112.7 ± 0.03	129.68 ± 7.56	112.04 ± 9.63

^*∗*^ *R*_*A*_ = average roughness.

^*∗∗*^ *R*_*Q*_ = root mean square roughness.

**Table 3:** ORR electrocatalytic parameters of graphite, PPy, and Mn/PPy catalysts obtained by RDE voltammograms (scan rate: 5 mV s^−1^) in O_2_-saturated 0.1 M KOH at 1600 rpm.

WE	*E*_onset_, V/RHE	*E*_1/2_, V/RHE	*i* at *E* = 0.5 V/RHE/mA cm^−2^	*n* at 0.8 V
Poly-Pt	0.871	0.72	4.12	3.79 ± 0.09
Graphite	0.82	0.7	1.78	2.21 ± 0.08
PPy on graphite (PPy/G)	0.85	0.69	2.16	2.74 ± 0.19
Mn-PPy on graphite (Mn-PPy60/G)	0.846	0.65	1	2.25 ± 0.18
Mn-PPy on graphite (Mn-PPy240/G)	0.9	0.714	1.65	4.08 ± 0.25
